# Key genes and integrated modules in hematopoietic differentiation of human embryonic stem cells: a comprehensive bioinformatic analysis

**DOI:** 10.1186/s13287-018-1050-7

**Published:** 2018-11-08

**Authors:** Pengfei Li, Mengyao Wu, Qiwang Lin, Shu Wang, Tong Chen, Hua Jiang

**Affiliations:** 1 Department of Hematology, Huashan Hospital, Fudan University, Shanghai, 200040 China; 2 Department of Gynecology, Obstetrics and Gynecology Hospital, Fudan University, Shanghai, 200011 China

**Keywords:** Embryonic stem cell, Differentiation, Hematopoietic stem cell, Microarray

## Abstract

**Background:**

The generation of hematopoietic stem cells (HSCs) and blood cells from human embryonic stem cells (hESCs) is a major goal for regenerative medicine; however, the differentiation mechanisms are largely undefined. Here, we aimed to identify the regulated genes and functional modules related to the early differentiation of the endothelial-to-hematopoietic transition (EHT) using comprehensive bioinformatics analyses.

**Methods:**

Undifferentiated hESCs (hESC-H9), CD34^+^ cells from 10-day differentiated hESC-H9 cells, and CD34^+^ cells from umbilical cord cells were isolated and collected. Cells from these three groups were subjected to RNA extraction and microarray analysis by which differentially expressed genes (DEGs) and time-series profiles were analyzed by significance analysis of microarray (SAM) and short time-series expression miner (STEM) algorithms. Gene enrichment analysis was performed by *ClusterProfiler* Package in *Rstudio*, while a protein-protein interaction (PPI) network was constructed by search tool for the retrieval of interacting genes (STRING) and visualized in Cytoscape. Hub genes were further identified with the MCODE algorithm in Cytoscape.

**Results:**

In the present study, we identified 11,262 DEGs and 16 time-series profiles that were enriched in biological processes of chromosome segregation, cell cycle, and leukocyte activation and differentiation, as well as hematopoiesis. Analysis using the MCODE algorithm further identified six integrated modules that might play an important role in the EHT process, including mitosis/cell cycle, mitochondrial process, splicing, ubiquitination, ribosome, and apoptosis.

**Conclusions:**

The study identified potential genes and integrated functional modules associated with the hematopoietic and endothelial differentiation of human ESCs.

**Electronic supplementary material:**

The online version of this article (10.1186/s13287-018-1050-7) contains supplementary material, which is available to authorized users.

## Background

Human embryonic stem cells (hESCs) and induced pluripotent stem cells (iPSCs), termed human pluripotent stem cells (hPSCs), are reported to be able to produce all lineages of blood cells with the potential for blood transfusion. However, the clinical application of hPSCs has been hampered due to the inefficiency of current differentiation procedures in generating hPSC-derived blood products [1]. To overcome this obstacle, dissecting the key regulators of hematopoietic differentiation has become a hotspot in hPSC research.

During embryogenesis, hemogenic endothelial (HE) cells, a specialized endothelial cell lineage, give rise to multipotent hematopoietic stem cells (HSCs) through a complex process designated the endothelial-to-hematopoietic transition (EHT) [2–5]. After induction with hematopoietic cytokines, human iPSCs are able to differentiate into CD34^+^ HE progenitors, which subsequently generate hematopoietic cells and mature endothelial cells, indicating that emergence of CD34^+^ HE cells from undifferentiated human iPSCs mimics the early embryonic EHT process in nature [6]. However, compared with adult CD34^+^ cells, the compromised capacity of human iPSC-derived CD34^+^ cells to proliferate, differentiate, and engraft highlights the differences in EHT in vitro and in vivo [7–9], in which both stem cells and the surrounding differentiating microenvironment are critical variables. Hence, the dynamic and precise control of gene expression needs clarification in the differentiated process [10].

**Fig. 1:**
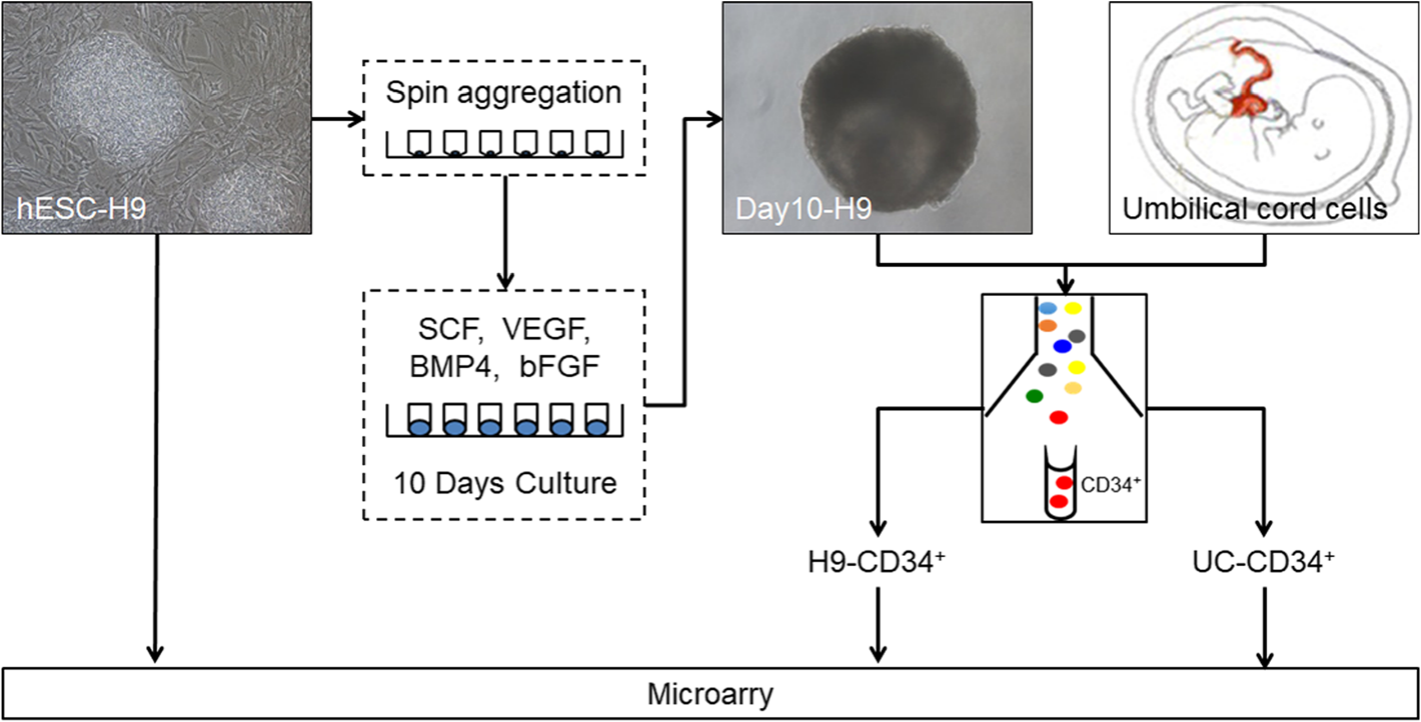
Flow chart of the comprehensive bioinformatic analysis. The undifferentiated hESC-H9, CD34^+^ cells derived from 10-day hematopoietic cytokine-stimulated differentiated hESC-H9 cells, and CD34^+^ cells from UC cells were employed, respectively. Cells in the above three groups were subjected to RNA extraction and microarray analysis

In the current work, we attempted to investigate the genetic alteration in ESCs-HE-HSCs transformation process by a time-series setting. The original undifferentiated hESC-H9 cells and CD34^+^ cells derived from 10-day hematopoietic cytokine-stimulated differentiated hESC-H9 cells were employed as the ESC-HE stage. Taken into the adult hematopoiesis, CD34^+^ cells in umbilical cord (UC) were selected as the control group of adult HSCs. All the cell groups were subject to RNA microarray analysis (Fig. 1). Through an integrative analysis that combined changes in functional gene expression into a genetic network, we identified six functional modules that might be related to the generation of CD34^+^ ECs from hESCs.

## Materials and methods

### Cell lines and cultivation

Human embryonic stem cell (ESC) line H9 (WiCell Research Institute, Madison, WI, http://www.wicell.org) was cultured as undifferentiated cells by passaging in a feeder-free culture system on Matrigel (BD Bioscience, Bedford, MA)-coated dish and using E8 serum-free medium (STEMCELL Technologies, Vancouver, Canada). The cells were passaged every 3–4 days by dissociation with ReleSR (STEMCELL Technologies, Vancouver, Canada).

Before differentiation, H9 cell media was changed from E8 medium to Dulbecco’s modified Eagle medium/nutrient mixture F-12 (DMEM/F12) complete medium prior to co-culture with irradiated (30 Gy) mouse embryonic fibroblasts (MEF). The DMEM/F12 complete medium consists of 20% knockout serum replacement (KSR), 1% non-essential amino acids, 1% mM l-glutamine (Invitrogen), 0.1 mM b-mercaptoethanol (Sigma Aldrich, Oakville, ON, Canada), and 5 ng/ml human basic fibroblast growth factor (bFGF, Peprotech, NJ). The cells were passaged weekly by dissociation with 0.05% trypsin.

### Formation of embryoid bodies

Undifferentiated H9 cells co-cultured with MEF were treated with 0.05% trypsin. embryoid body (EB) formation was performed using forced aggregation in a spin EB medium based on serum-free medium (SFM) and supplemented with 20 ng/ml human bone morphogenetic protein 4 (BMP4), 50 ng/ml human stem cell factor (SCF), 20 ng/ml human vascular endothelial growth factor (VEGF), and 10 ng/ml bFGF. The cell suspension was placed in 96-well low-attachment plates at 100 μl/well, centrifuged at 1500 rpm for 5 min. EBs were cultured for 10 days with media changes every 3 days.

### Flow cytometry and fluorescence-activated cell sorting

Single-cell suspensions of EBs differentiated at day 10 were prepared as follows: EBs were dissociated with 0.05% trypsin for 10 min in a 37 °C water bath. For flow cytometry, cells were resuspended at approximately 1.0 × 10^5^ cells/ml with PBS containing 2% FBS and stained with fluorochrome-conjugated monoclonal antibodies including fluorescein isothiocyanate (FITC)- and phycoerythrin (PE)-conjugated CD34, allophycocyanin (APC)-conjugated CD31, and PE-conjugated VE-cadherin, peridinin chlorophyll protein complex (PerCP)-conjugated CD45 at a concentration of 5 μg/ml. Cells were stained for 30 min at 4 °C and washed in PBS containing 2% FBS. For isolation of CD34^+^ cells, dissociated EB cells or mononuclear UC cells were stained with PE-conjugated CD34 and isolated by FACS with a FACSAria III (BD Biosciences, Franklin Lakes, NJ). Hematopoietic colony-forming assays of H9-CD34^+^ cells were performed according to the protocol of MethoCult™ H4434 Classic (STEMCELL Technologies, Vancouver, Canada).

### RNA extraction and microarray experiments

For the global transcriptional analysis, total RNA was extracted from undifferentiated hESC-H9, H9-CD34^+^ cells, and UC-CD34^+^ cells using TRIzol reagent (Invitrogen, Carlsbad, CA, USA) according to the manufacturer’s instructions. The genome-wide gene expression profiling was performed using the Human Transcriptome Array 2.0 (HTA 2.0, Affymetrix, USA). Genes with fold values between − 2 and + 2 were omitted from the analysis. Data from three comparisons were analyzed, namely hESC-H9 cells vs H9-CD34^+^ cells, hESC-H9 vs UC-CD34^+^ cells, and H9-CD34^+^ cells vs UC-CD34^+^ cells. The mRNA expression microarray data was extracted by the *affy* package in *Rstudio* [11]. The robust multiarray average (RMA) method was used to normalize the data through log2 transformation.

### Differentially expressed genes analysis

Taking into consideration the sample size and the three comparisons in our experiments, significance analysis of microarray (SAM) analysis based on *Siggenes* Package in *Rstudio* was applied to analyze the differentially expressed genes [12]. Fold change and false discovery rate (FDR) were the major indexes for the differentially methylated gene (DEG) screen in a two-group setting. In here, fold change was not available, and genes with FDR ≤ 0.01 were noted as DEGs. The relationships between samples and DEGs were shown by hierarchical clustering heatmaps.

### Time-series expression analysis

Short time-series expression miner (STEM) program is a Java-based software specifically designed for the analysis of short time-series microarray gene expression data [13]. Here, undifferentiated hESC-H9, H9-CD34^+^ cells, and UC-CD34^+^ cells were set as time series, which started at undifferentiated hESC-H9. Normalized DEGs data in each biological repetition was entered into the program, with all parameter settings to the default value. Each gene was assigned to the closest profile by the correlation coefficient. We performed the permutation-based test to quantify the expected number of genes that would be assigned to each profile. The *p* value derived from STEM analysis was adjusted for multiple hypothesis testing, using a *q* value < 0.05. The profile boxes were colored if statistically significant. The most significant upregulated or downregulated profiles were subjected to further analysis.

### Gene ontology enrichment and Kyoto Encyclopedia of Genes and Genomes pathway analysis

Gene Ontology (GO) enrichment and Kyoto Encyclopedia of Genes and Genomes (KEGG) pathway analysis were performed by over-representation analysis (ORA) with Fisher’s exact test, and Benjamini-Hochberg (B-H) multiple test correction method was used to correct for the occurrence of false positives. A strict cutoff of *p* values < 0.01 and FDR < 0.05 was used. The statistics and data visualization were performed by *ClusterProfiler* Package in *Rstudio* [14]. The 16 most significant terms of each module were noted.

### Construction of protein-protein interaction network

The potential protein interaction was predicted based on the search tool for the retrieval of interacting genes (STRING) database [15]. The minimum required interaction score was set as 0.4 by default. The network visualization was performed by Cytoscape software version 3.5.1 (http://www.cytoscape.org/).

We further performed MCODE clustering analysis to identify molecular modules in the protein-protein interaction (PPI) network [16]. The parameter settings included a degree cutoff ≥ 2 to prevent an artificially high node score, and a *k*-core value ≥ 4 to exclude relatively small clusters. CytoHubba, another plug-in program, was applied to the hub genes screen with the recommended maximal clique centrality (MCC) ranking methods [17]. The first three genes were noted as hub genes.

## Results

Undifferentiated H9 cells closely resembled the growth of colony-like adherent cells (Fig. 1). Due to that CD34 is a marker being consistently expressed on HE cells and HSCs [18], we sorted CD34^+^ cells from 10-day hematopoietic cytokine-stimulated differentiated hESC-H9 cells and UC to investigate the genetic alternation at different differentiating stages.

**Fig. 2:**
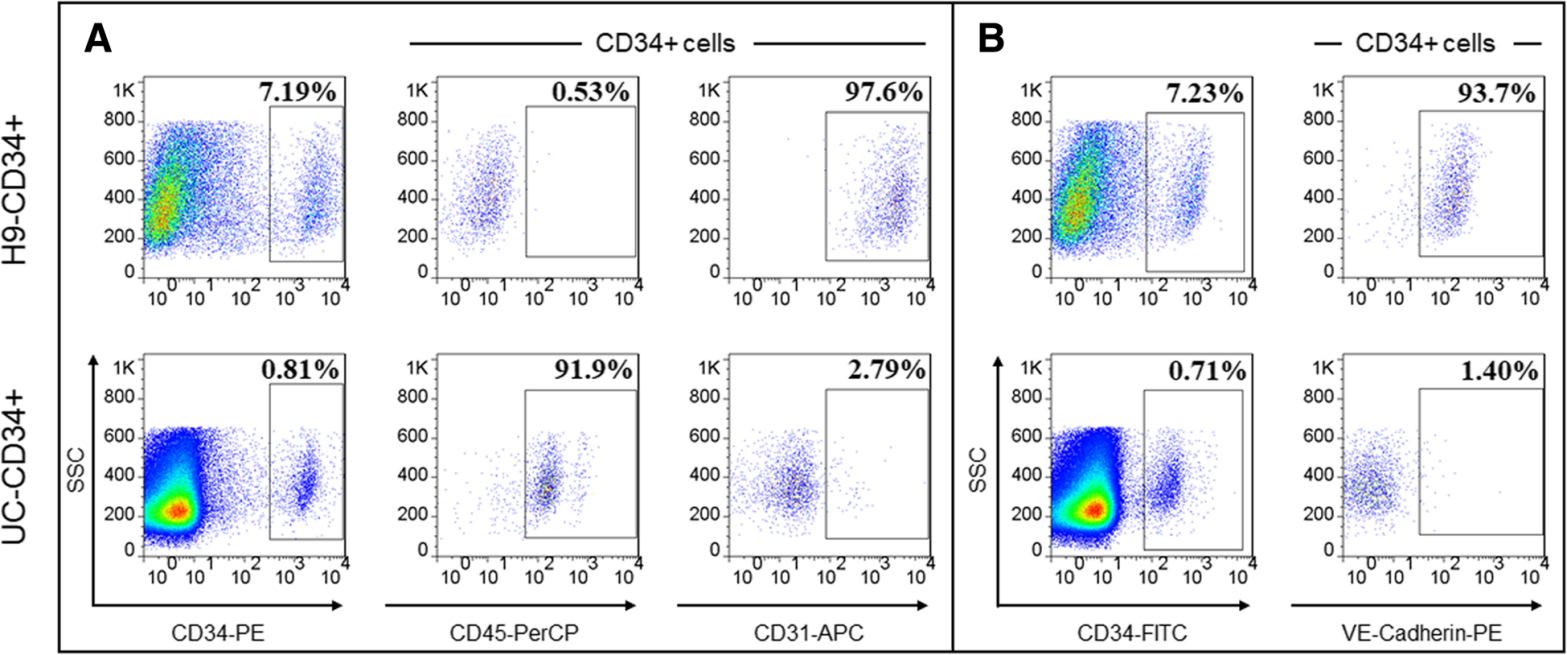
Flow cytometric analysis. Flow cytometric analysis of CD45, CD31, and VE-cadherin expression on CD34^+^ cells derived from hESC-H9 cells and CD34^+^ cells from UC cells. In which, PE-coagulated CD34, PerCP-coagulated CD45, and APC-coagulated CD31 were performed in the same condition (**a**). FITC-coagulated CD34 and PE-coagulated VE-cadherin were performed in another identical condition (**b**)

As shown in the top panel of Fig. 2a, b, H9-CD34^+^ cells co-expressed endothelial markers CD31 (97.6%) and VE-cadherin (93.7%) and additionally were able to give rise to hematopoietic colonies (Additional file 1: Figure S1). Taking these together, it was shown that H9-CD34^+^ cells exhibited HE cell features with endothelial and hematopoietic potential. However, UC-CD34^+^ cells showed CD45^+^CD31^low^VE-Cadherin^low^ pattern (Fig. 2a, b), indicating the increasingly hematopoietic and decreasingly endothelial characteristics.

**Fig. 3:**
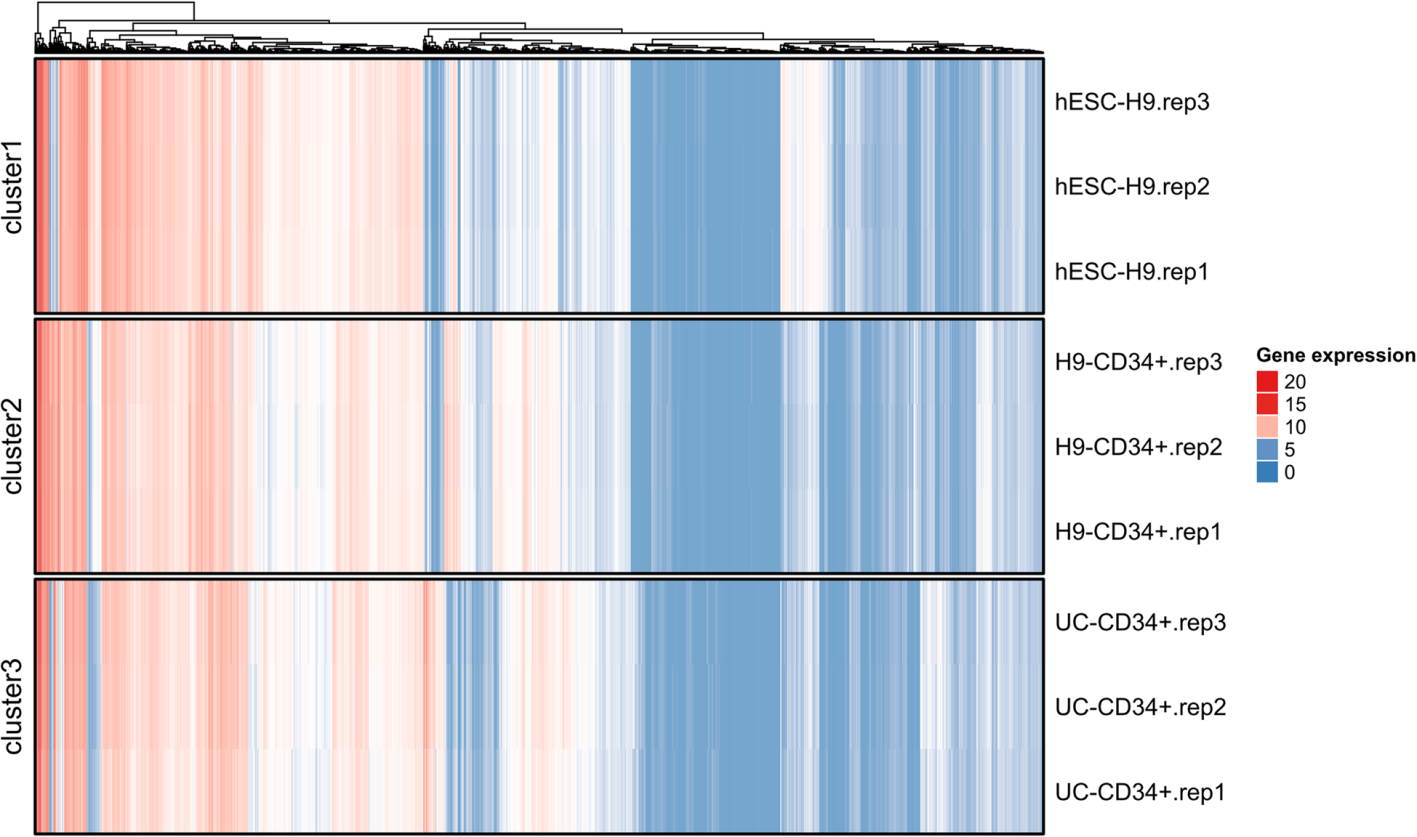
Heatmap of the DEGs. Log2 normalized microarray data was subjected to SAM analysis, and 11,262 DEGs were identified. The samples were clustered and identified as individual time series (hESC-H9, H9-CD34^+^, UC-CD34^+^), as shown in the heatmap. Rows indicate individual genes, and columns indicate the three groups (with three biological replicates for each). The color scale bar indicates the log2 expression ratio (red = upregulated; green = downregulated)

The original microarray expression data are shown in Additional file 2: Table S1. After SAM analysis, we identified 11,262 DEGs in the undifferentiated hESC-H9 cells, differentiated H9-derived CD34^+^ cells at day 10 (H9-CD34^+^), and umbilical cord blood-derived CD34^+^ cells (UC-CD34^+^). As shown in the hierarchical clustering analysis heatmap (Fig. 3), the samples were clustered as different time series (hESC-H9, H9-CD34^+^, and UC-CD34^+^), which indicated that the group setting was reasonable and ready for further analysis.

**Fig. 4:**
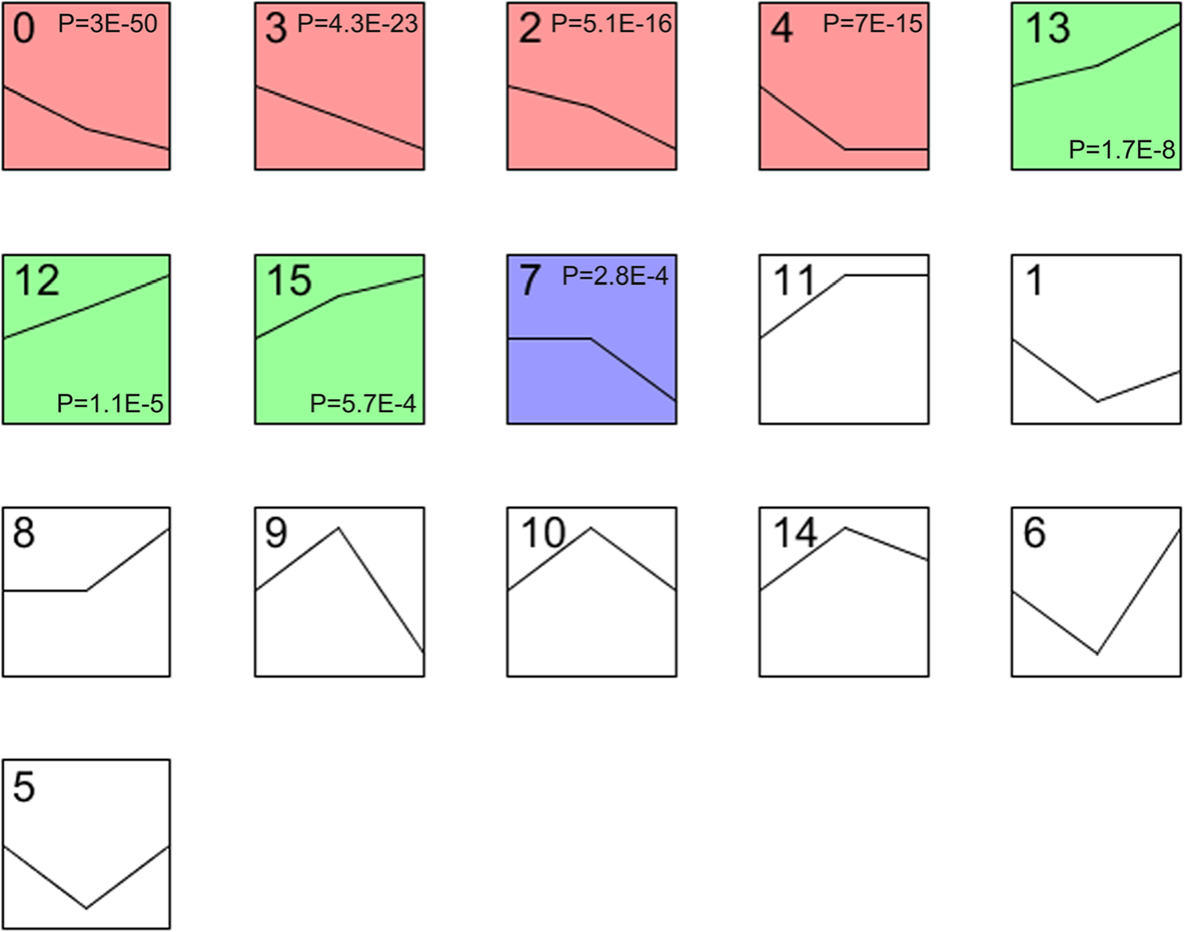
STEM analysis of the gene profiles with different expression patterns. The number in the top left-hand corner of a profile box is the profile ID number, assigned by STEM according to the expression tendency. The colored profiles had a statistically significant number of genes assigned, with *p* value labeled in the top or bottom right-hand corner. Non-white profiles of the same color represent profiles with the similar tendency, with the *p* value omitted. In each profile box, the black lines showed model expression profiles, and the *x*-axis represented the different time series (hESC-H9, H9-CD34^+^, and UC-CD34^+^). The time series was log-normalized to start at hESC-H9. The *y*-axis of all genes assigned a profile box were at the same scale

In the STEM analysis, using undifferentiated hESC-H9 as a reference, 16 profiles were identified according to the expression tendency (Fig. 4). Among them, statistically significant *p* values were found in profiles 0, 3, 2, 4, 13, 12, 15, and 7. Genes in the first four profiles (0, 3, 2, 4) decreased consistently but increased in the profiles of 13, 12, and 15. Genes in profile 7 remained unchanged and declined only after 10 days of differentiation.

**Fig. 5:**
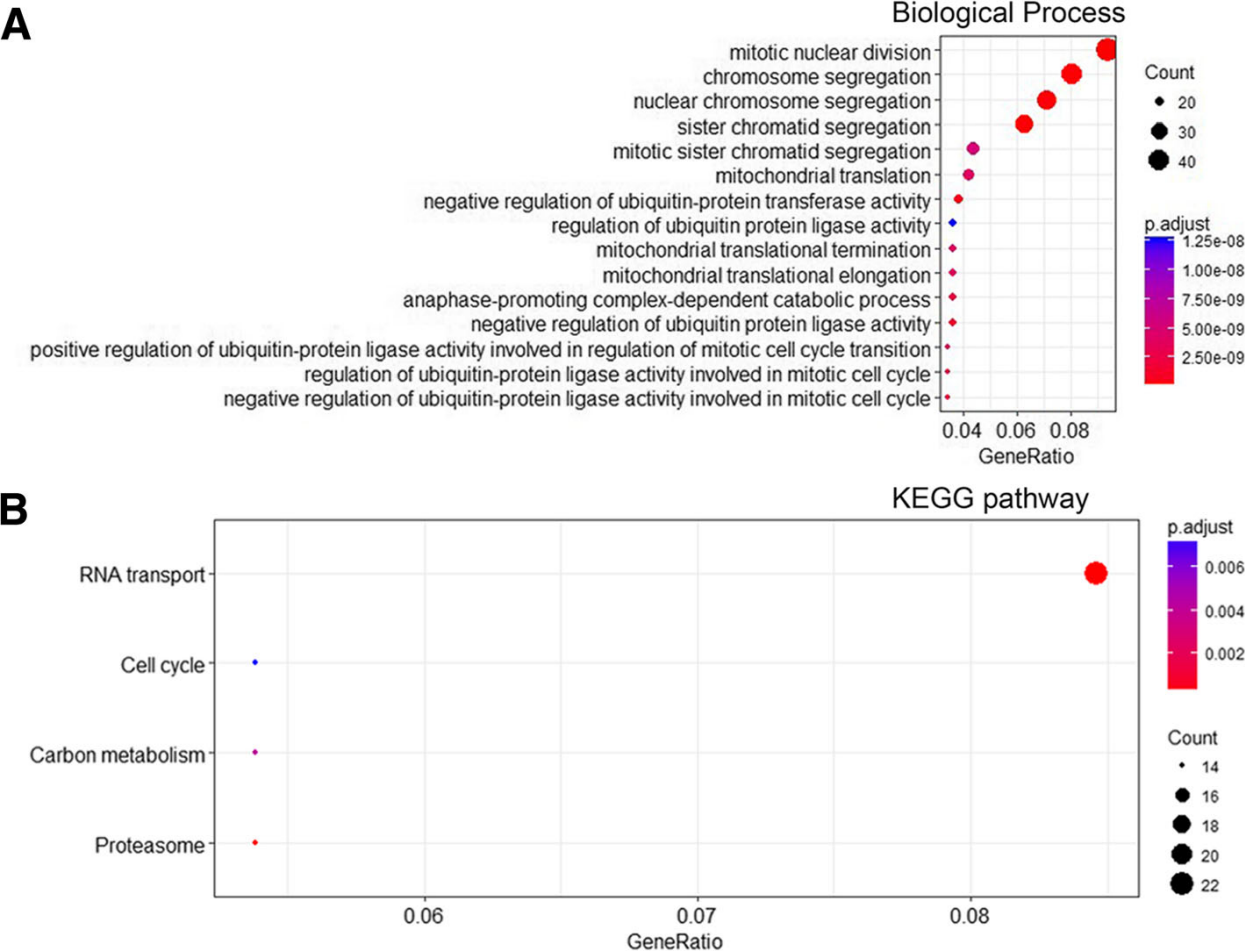
The GO enrichment and KEGG pathway analysis in profile 0. The top 15 biological processes (**a**) and four KEGG pathways (**b**) were shown. Three parameters including gene ratio, gene counts, and adjusted *p* value were used to evaluate the enriched items

**Fig. 6:**
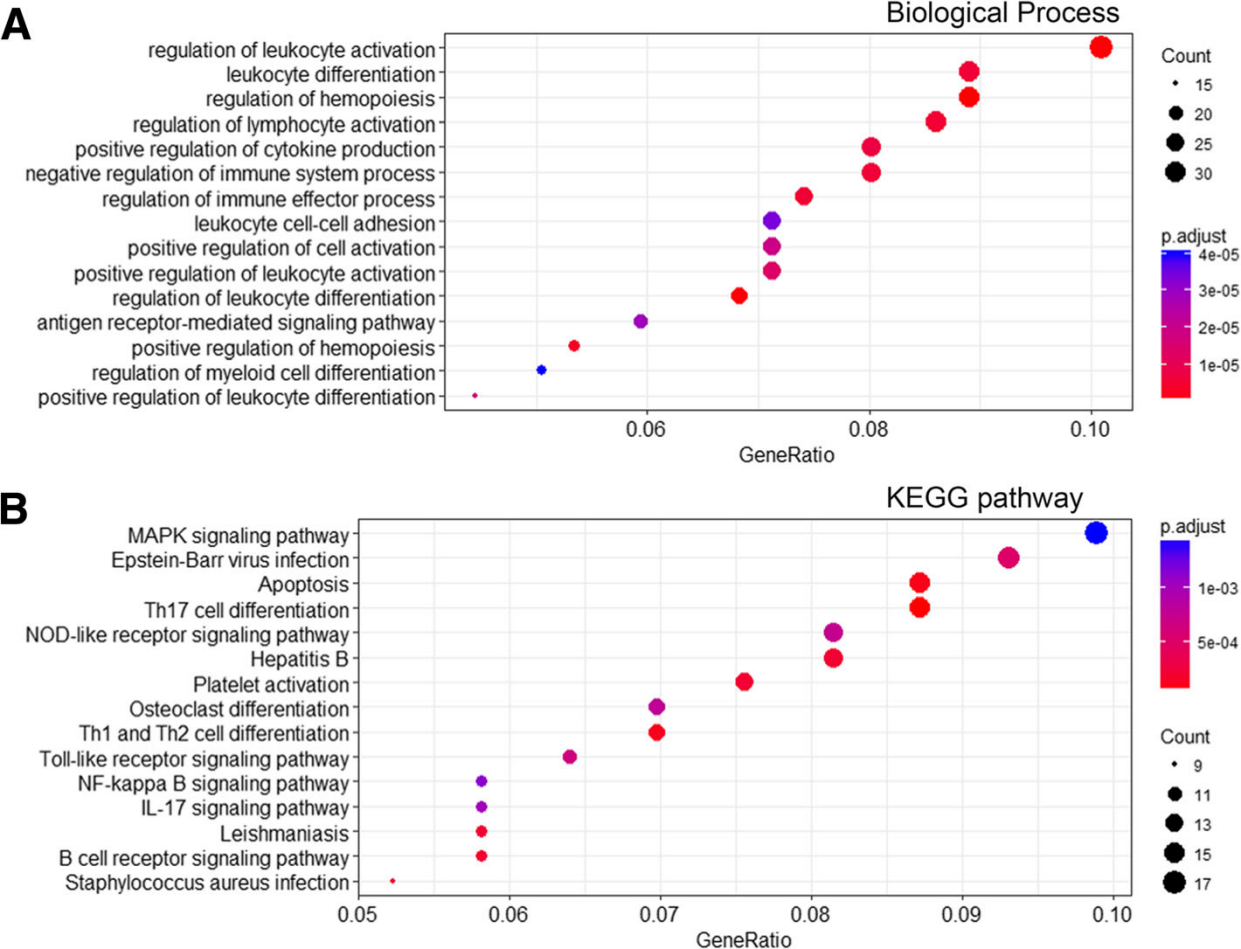
The GO enrichment and KEGG pathway analysis in profile 13. The top 15 biological processes (**a**) and KEGG pathways (**b**) were shown. Three parameters including gene ratio, gene counts, and adjusted *p* value were used to evaluate the enriched items

To investigate the biological functions in the different profiles, we selected the most significantly downregulated profile 0 and upregulated profile 13 for the GO and KEGG analyses. As shown in Fig. 5a, the majority of genes in profile 0 were associated with cell mitosis and ubiquitination, while four pathways associated with RNA transport, cell cycle, carbon metabolism, and proteasome were also enriched (Fig. 5b). In addition, the leukocyte functional and differentiation regulation pathway was enriched in profile 13 and is shown in Fig. 6a. Associated pathways, such as the inflammatory and immunological pathways, were diverse but consistent with the biological process (Fig. 6b). The number of genes and *p* values are shown in Additional file 3: Table S2.

**Fig. 7:**
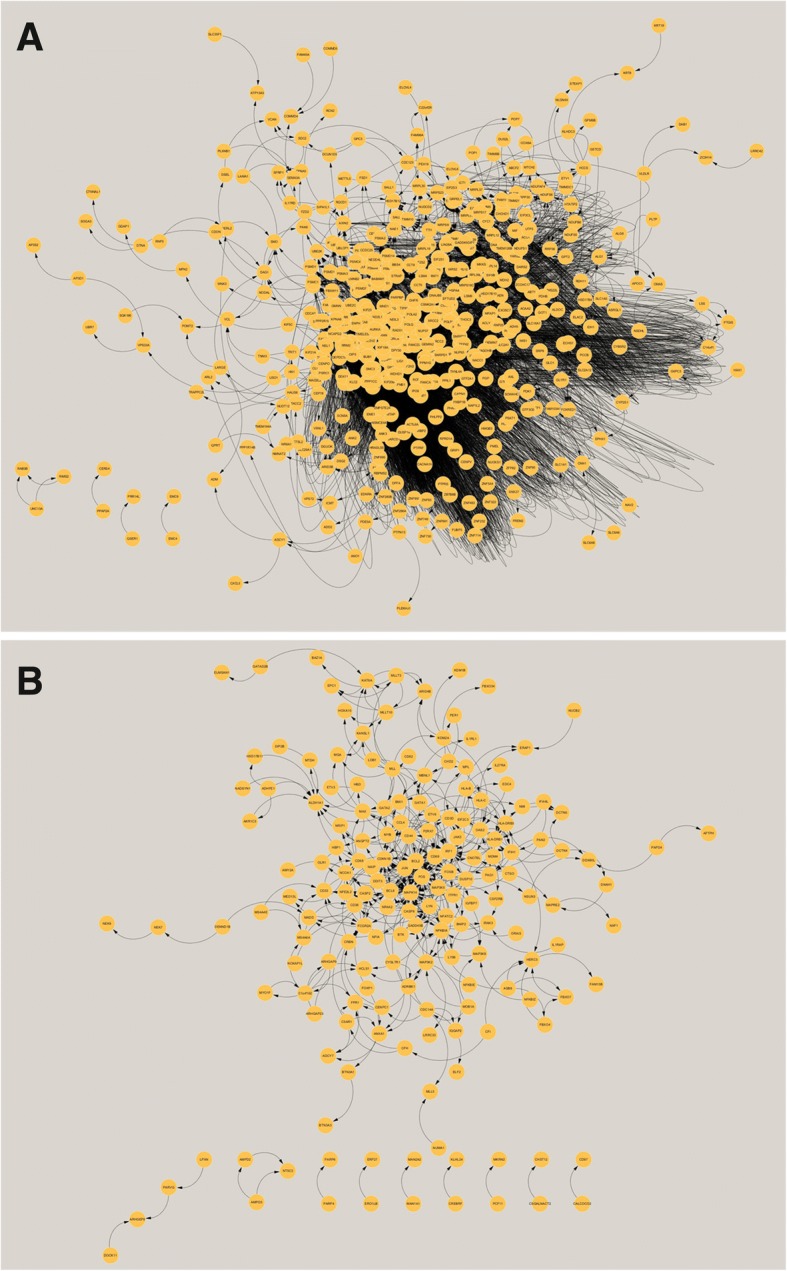
PPI network analysis. A PPI network was visualized by Cytoscape, **a** for profile 0 and **b** for profile 13. The yellow circles indicate the genes identified by the Cytoscape program. Black arrows indicate connections between genes

**Fig. 8:**
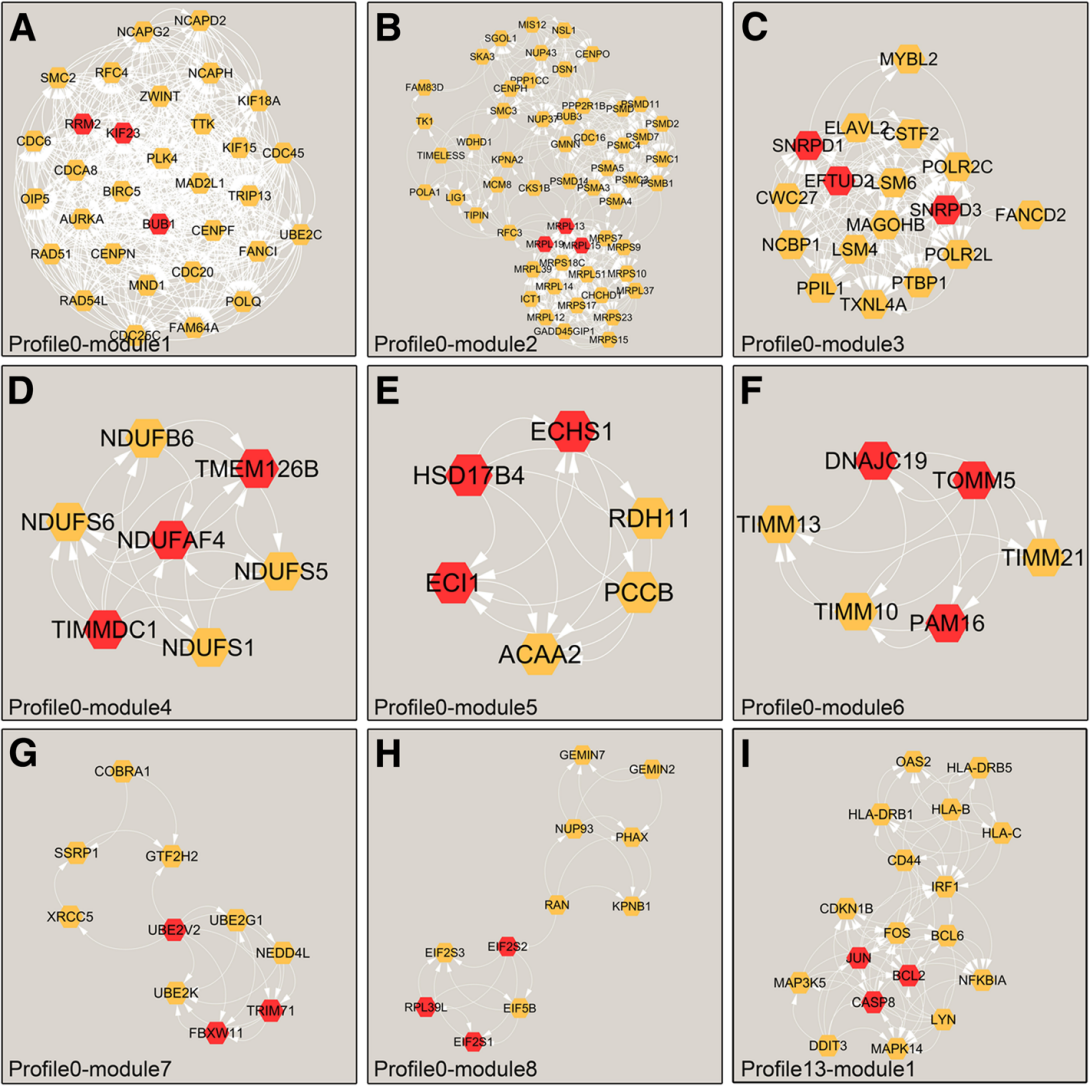
Modules identified by MCODE algorithm in Cytoscape for the two profiles. Profile 0 was classified into eight modules (**a**-**h**), and profile 13 was classified into only one module (**i**). As shown in each module, the red box indicates the hub genes identified by the CytoHubba program

**Fig. 9:**
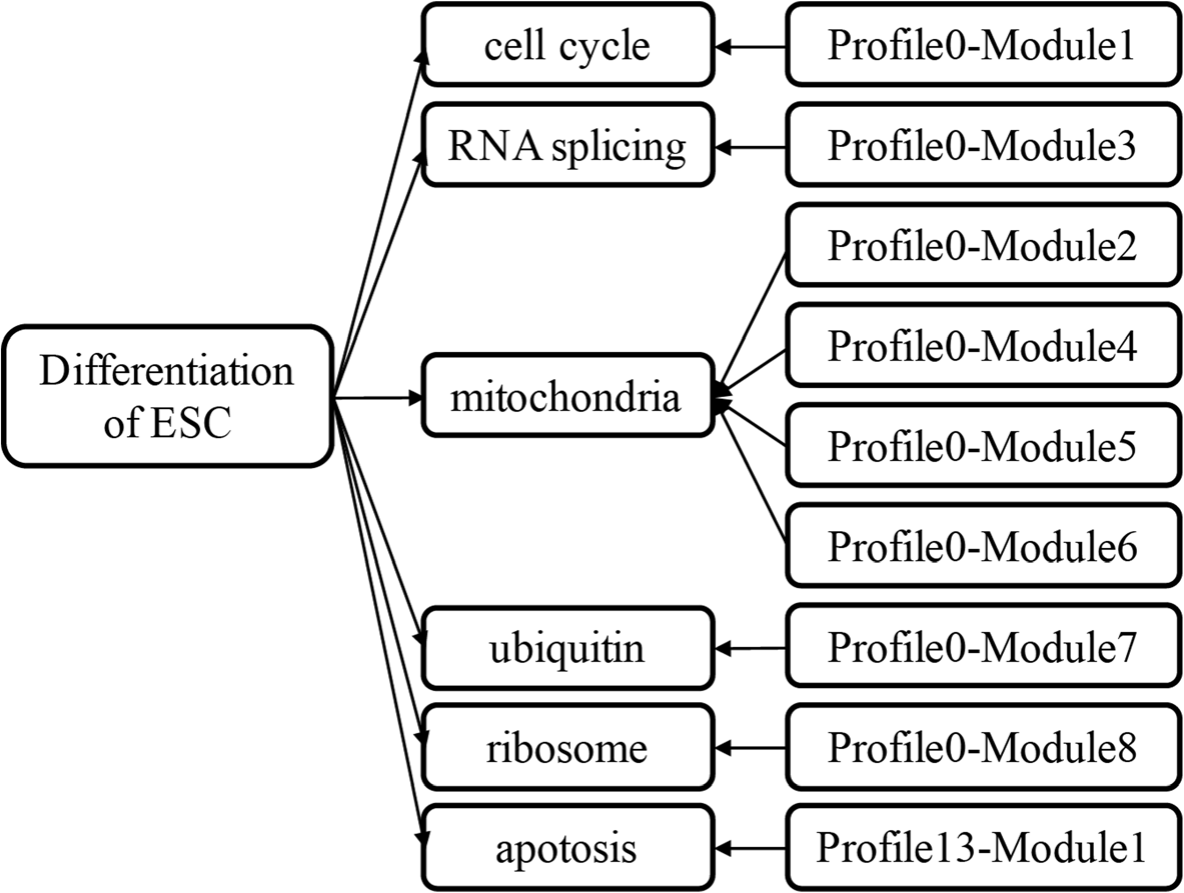
The six integrated modules and functional annotations. The most significantly upregulated profile 13 and downregulated profile 0 were further investigated. Six modules were involved with various biological processes relating to mitosis/cell cycle, mitochondrial process, splicing, ubiquitination, ribosome, and apoptosis

PPI was calculated theoretically and simulated using the online STRING database. Data were visualized by Cytoscape and are shown in Fig. 7. In total, 462 nodes and 3241 edges were detected in downregulated profile 0, and 173 nodes and 404 edges were found in upregulated profile 13 (Fig. 8). With the MCODE algorithm, 8 core modules and 1 core module were found in profiles 0 and 13, respectively (Fig. 9). Hub genes in each module are shown in Additional file 4: Table S3. Among the modules in downregulated profile 0, RRM2, BUB1, and KIF23 of module 1 were proven to be involved in cell mitosis and the cell cycle. MRPL13/15/19 of module 2; NDUFAF4, TMEM126B, and TIMMDC1 of module 4; ECHS1, HSD17B4, and ECI1 of module 5; and TOMM5, PAM16, and DNAJC19 of module 6 are all key elements associated with mitochondria, such as the synthesis of proteins in the mitochondria, respiratory chain complex I assembly, mitochondrial fatty acid beta-oxidation pathway, and the mitochondrial translational. In addition, SNRPD3, SNRPD1, and EFTUD2 of module 3 are associated with spliceosome complex assembly in RNA transcription. UBE2V2, UBE2G1, and NEDD4L of module 7 are involved with protein ubiquitination, and in module 8, EIF2S2 and RPL39L are related to organ development. While, in module 1 of the upregulated profile 13, bcl-2 and caspase8, which are well-known regulators of apoptosis, were present.

Finally, according to the functional annotations in NCBI, all of the nine modules of profiles 0 and 13 were artificially classified into six integrated functional modules (Fig. 9). Modules 2, 4, 5, and 6 from profile 0 were termed mitochondrial process module, while modules 1, 3, 7, and 8 in profile 0 and module 1 in profile 13 were termed mitosis/cell cycle module, splicing module, ubiquitination module, ribosome module, and apoptosis module, respectively.

## Discussion

In recent years, many efforts have been made to improve the efficiency of generating blood products from hPSCs. During hematopoiesis, CD34^+^ HE cells have been identified as the precursors of multipotent HSCs that give rise to hematopoietic and endothelial progeny through the EHT. In this study, using an in vitro differentiation model, we focused on differential gene expression during hematopoiesis using comprehensive biological and bioinformatics analyses. The identified time-series profiles and modules will help determine the genes regulating hematopoietic differentiation by hPSCs.

Considering the dynamic changes in gene expression during hematopoiesis, we applied the STEM program to identify and further characterize eight significant time-series profiles (0, 2, 3, 4, 7, 12, 13, and 15, *p* < 0.05). The most significantly upregulated profile 13 and downregulated profile 0 were further investigated. The enrichment of genes in these two profiles demonstrated the key biological processes in the differentiation of HSCs.

The hESC fate in apoptosis, proliferation, and differentiation is orchestrated by controlling the expression of a series of genes. In upregulated profile 13, the majority of enriched genes were related to hematopoiesis, which is consistent with hematopoietic differentiation in nature. Interestingly, the PPI network of profile 13 indicated that Jun, caspase8, and bcl-2 were identified as hub genes. Downregulation of Jun is required to maintain the ground-state pluripotency of ESC [19]. The bcl-2 family mediates intrinsic “mitochondria development” apoptotic signaling, while the caspase family is related to the extrinsic “death receptor-mediated” apoptotic pathway [20], both of which are conserved cascade pathways that regulate apoptosis and programmed cell death. Furthermore, bcl-2 and caspase8 have been documented to mediate a non-apoptotic process in embryogenesis and hematopoiesis [21, 22]. In murine ESCs, bcl-2 enhanced hematopoietic cell differentiation by accelerating EB formation [23]. In lymphocyte clonal expansion, the catalytically competent form of caspase8 maintained T cell proliferation, which was independent from apoptosis [21, 24, 25]. Our data consistently indicated that the non-apoptotic function of bcl-2 and caspase8 in hematopoietic differentiation of hESCs might be regulated together with the Jun-associated pathway.

In the downregulated profile 0, three of the five integrated modules, including mitotic/cell cycle, RNA splicing, and ubiquitination process were the common mechanisms in the development of ESCs [26–28]. The mitochondrial process and ribosomal modules have not been previously reported. In our data, the hub genes of modules 6, 2, 4, and 5 were related to the transport of mitochondrial proteins, mitochondrial ribosomes, and energy metabolism of mitochondria. Thus, these four modules were collectively termed as a single mitochondrial-associated module. Together, they highlight a new dimension of mitochondrial function in stem cell biology. In ribosomal module 8 of profile 0, all three hub genes, EIF2S2, EIF2S1, and RPL39L, were associated with the initiation of eukaryotic gene translation by association with the ribosome. Although it is not known whether the encoded protein was a functional ribosomal protein or indirectly associated with the ribosome, it has been reported that ribosomal proteins not only have structural functions but also impact mouse ESC differentiation [29].

## Conclusions

In summary, our study identified several potential genes associated with hematopoietic differentiation of hESCs. Among them, six modules were involved with various biological processes relating to mitosis/cell cycle, mitochondrial process, splicing, ubiquitination, ribosome, and apoptosis. Future studies are needed to not only identify the gene functions in different modules but also dissect the regulatory gene networks during early hematopoietic development and identify the requirements to improve the efficiency of generating hPSC-derived hematopoietic products for regenerative medicine.

## Additional files

Additional file 1: **Figure S1.** H9-CD34^+^ cells were able to form hematopoietic colonies. Colony-forming assays were performed by culturing H9-CD34^+^ cells in MethoCult™ H4434 Classic media. Colonies were clarified as CFU-GM (colony forming unit granulocyte, macrophage), CFU-M (colony-forming unit macrophage) and CFU-G (colony-forming unit granulocyte) after 2 weeks. The scale bar indicated 100 μm (bright-field, orig. mag. × 10). (TIF 1515 kb)

Additional file 2: **Table S1.** The normalized expression data in the microarray datasets. (XLSX 1769 kb)

Additional file 3: **Table S2.** The number of genes and *p* value in sixteen profiles. (XLSX 33 kb)

Additional file 4: **Table S3.** Hub genes in nine core modules in profiles 0 and 13. (XLSX 46 kb)

## Abbreviations

APC: Allophycocyanin
B-H: Benjamini-Hochberg
DEG: Differentially expressed gene
DMEM/F12: Dulbecco’s modified Eagle medium/nutrient mixture F-12
EB: Embryoid body
EHT: Endothelial-to-hematopoietic transition
FACS: Fluorescence-activated cell sorting
FDR: False discovery rate
FITC: Fluorescein isothiocyanate
GO: Gene Ontology
HE: Hemogenic endothelial
hESCs: Human embryonic stem cells
hPSCs: Human pluripotent stem cells
HSCs: Hematopoietic stem cells
iPSCs: Induced pluripotent stem cells
KEGG: Kyoto Encyclopedia of Genes And Genomes
KSR: Knockout serum replacement
MCC: Maximal clique centrality
MEF: Mouse embryonic fibroblast
ORA: Over-representation analysis
PE: Phycoerythrin
PerCP: Peridinin chlorophyll protein complex
PPI: Protein-protein interaction
RMA: Robust multiarray average
SAM: Significance analysis of microarray
STEM: Short time-series expression miner

## References

[1] Bordignon C (2006). Stem-cell therapies for blood diseases. Nature.

[2] Jaffredo T, Gautier R, Eichmann A, Dieterlen-Lievre F (1998). Intraaortic hemopoietic cells are derived from endothelial cells during ontogeny. Development.

[3] Zovein AC, Hofmann JJ, Lynch M, French WJ, Turlo KA, Yang Y (2008). Fate tracing reveals the endothelial origin of hematopoietic stem cells. Cell Stem Cell.

[4] Bertrand JY, Chi NC, Santoso B, Teng S, Stainier DY, Traver D (2010). Haematopoietic stem cells derive directly from aortic endothelium during development. Nature.

[5] Kissa K, Herbomel P (2010). Blood stem cells emerge from aortic endothelium by a novel type of cell transition. Nature.

[6] Chadwick K, Wang L, Li L, Menendez P, Murdoch B, Rouleau A (2003). Cytokines and BMP-4 promote hematopoietic differentiation of human embryonic stem cells. Blood.

[7] Woods NB, Parker AS, Moraghebi R, Lutz MK, Firth AL, Brennand KJ (2011). Brief report: efficient generation of hematopoietic precursors and progenitors from human pluripotent stem cell lines. Stem Cells.

[8] Wang L, Menendez P, Shojaei F, Li L, Mazurier F, Dick JE (2005). Generation of hematopoietic repopulating cells from human embryonic stem cells independent of ectopic HOXB4 expression. J Exp Med.

[9] Dou DR, Calvanese V, Sierra MI, Nguyen AT, Minasian A, Saarikoski P (2016). Medial HOXA genes demarcate haematopoietic stem cell fate during human development. Nat Cell Biol.

[10] Kaufman DS, Hanson ET, Lewis RL, Auerbach R, Thomson JA (2001). Hematopoietic colony-forming cells derived from human embryonic stem cells. Proc Natl Acad Sci U S A.

[11] Gautier L, Cope L, Bolstad BM, Irizarry RA (2004). affy--analysis of Affymetrix GeneChip data at the probe level. Bioinformatics.

[12] Tusher VG, Tibshirani R, Chu G (2001). Significance analysis of microarrays applied to the ionizing radiation response. Proc Natl Acad Sci U S A.

[13] Ernst J, Bar-Joseph Z (2006). STEM: a tool for the analysis of short time series gene expression data. BMC Bioinformatics.

[14] Yu G, Wang LG, Han Y, He QY (2012). clusterProfiler: an R package for comparing biological themes among gene clusters. OMICS.

[15] Jeanquartier F, Jean-Quartier C, Holzinger A (2015). Integrated web visualizations for protein-protein interaction databases. BMC Bioinformatics.

[16] Bader GD, Hogue CW (2003). An automated method for finding molecular complexes in large protein interaction networks. BMC Bioinformatics.

[17] Chin CH, Chen SH, Wu HH, Ho CW, Ko MT, Lin CY (2014). cytoHubba: identifying hub objects and sub-networks from complex interactome. BMC Syst Biol.

[18] Zhang Y, Wang C, Wang L, Shen B, Guan X, Tian J (2017). Large-scale ex vivo generation of human red blood cells from cord blood CD34(+) cells. Stem Cells Transl Med.

[19] Veluscek G, Li Y, Yang SH, Sharrocks AD (2016). Jun-mediated changes in cell adhesion contribute to mouse embryonic stem cell exit from ground state pluripotency. Stem Cells.

[20] Delbridge AR, Grabow S, Strasser A, Vaux DL (2016). Thirty years of BCL-2: translating cell death discoveries into novel cancer therapies. Nat Rev Cancer.

[21] Leverrier S, Salvesen GS, Walsh CM (2011). Enzymatically active single chain caspase-8 maintains T-cell survival during clonal expansion. Cell Death Differ.

[22] Winkler IG, Bendall LJ, Forristal CE, Helwani F, Nowlan B, Barbier V (2013). B-lymphopoiesis is stopped by mobilizing doses of G-CSF and is rescued by overexpression of the anti-apoptotic protein Bcl2. Haematologica.

[23] Wang YY, Deng X, Xu L, Gao F, Flagg T, May WS (2008). Bcl2 enhances induced hematopoietic differentiation of murine embryonic stem cells. Exp Hematol.

[24] Maelfait J, Beyaert R (2008). Non-apoptotic functions of caspase-8. Biochem Pharmacol.

[25] Kubota K, Kato S, Akiyama T, Fujita K, Yoneda M, Takahashi H (2009). A proposal for differentiation between early- and advanced-stage autoimmune pancreatitis by endoscopic ultrasonography. Dig Endosc.

[26] Li VC, Kirschner MW (2014). Molecular ties between the cell cycle and differentiation in embryonic stem cells. Proc Natl Acad Sci U S A.

[27] Kim YD, Lee J, Kim HS, Lee MO, Son MY, Yoo CH (2017). The unique spliceosome signature of human pluripotent stem cells is mediated by SNRPA1, SNRPD1, and PNN. Stem Cell Res.

[28] Nguyen DTT, Richter D, Michel G, Mitschka S, Kolanus W, Cuevas E (2017). The ubiquitin ligase LIN41/TRIM71 targets p53 to antagonize cell death and differentiation pathways during stem cell differentiation. Cell Death Differ.

[29] Fortier S, MacRae T, Bilodeau M, Sargeant T, Sauvageau G (2015). Haploinsufficiency screen highlights two distinct groups of ribosomal protein genes essential for embryonic stem cell fate. Proc Natl Acad Sci U S A.

