# Supplementary figures and images for: Key genes and integrated modules in hematopoietic differentiation of human embryonic stem cells: a comprehensive bioinformatic analysis
Source: Stem Cell Res Ther. 2018 Nov 8;9:301. doi: 10.1186/s13287-018-1050-7 (PMC6225692; doi:10.1186/s13287-018-1050-7)

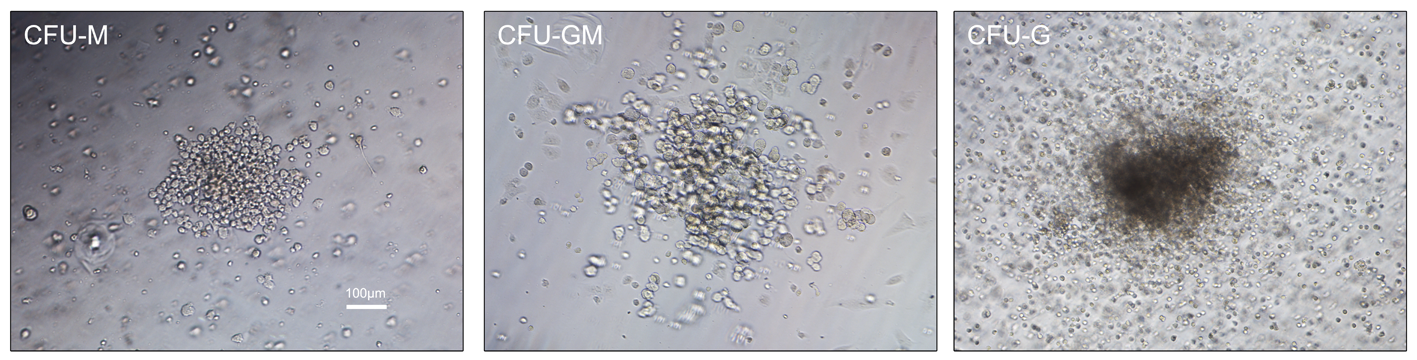

Supplement: Supplementary file 1 — Figure S1. H9-CD34+ cells were able to form hematopoietic colonies. Colony-forming assays were performed by culturing H9-CD34+ cells in MethoCult™ H4434 Classic media. Colonies were clarified as CFU-GM (colony forming unit granulocyte, macrophage), CFU-M (colony-forming unit macrophage) and CFU-G (colony-forming unit granulocyte) after 2 weeks. The scale bar indicated 100 μm (bright-field, orig. mag. × 10). (TIF 1515 kb) [file 13287_2018_1050_MOESM1_ESM.tif]
